# Heavy grazing reduced the spatial heterogeneity of *Artemisia frigida* in desert steppe

**DOI:** 10.1186/s12870-022-03712-8

**Published:** 2022-07-13

**Authors:** Zihan Wang, Shijie Lv, Guodong Han, Zhongwu Wang, Zhiguo Li, Haiyan Ren, Jing Wang, Hailian Sun, Guogang Zhang

**Affiliations:** 1 College of Grassland, Resources and Environment, Inner Mongolia Agricultural University, Hohhot, 010019 Inner Mongolia People’s Republic of China; 2 Science College, Inner Mongolia Agricultural University, Hohhot, 010018 Inner Mongolia People’s Republic of China; 3 Inner Mongolia Academy of Agricultural and Animal Husbandry Sciences, Hohhot, 010010 Inner Mongolia People’s Republic of China; 4 College of Life Sciences, Tianjin Normal University, 300387 Tianjin, People’s Republic of China

**Keywords:** Geo-statistics, Spatial distribution, Different scales

## Abstract

**Background:**

Grazing disturbance plays an important role in the desert steppe ecosystem in Inner Mongolia, China. Previous studies found that grazing affected the spatial distribution of species in a community, and showed patchiness characteristics of species under different grazing treatments. *Artemisia frigida* is the dominant species and semi-shrub in desert steppe, and whether grazing interference will affect the spatial distribution of *A. frigida* is studied. In this study, geo-statistical methods were mainly used to study the spatial distribution characteristics of *A. frigida* population in desert steppe of Inner Mongolia at two scales (quadrat size 2.5 m × 2.5 m, 5 m × 5 m) and four stocking rates (control, CK, 0 sheep·ha^–1^·month^–1^; light grazing, LG, 0.15 sheep·ha^–1^·month^–1^, moderate grazing, MG, 0.30 sheep·ha^–1^·month^–1^, heavy grazing, HG, 0.45 sheep·ha^–1^·month^–1^).

**Results:**

The results showed that the spatial distribution of *A. frigida* tended to be simplified with the increase of stocking rate, and tended to be banded with increased spatial scale. The density and height of *A. frigida* increased with increasing scale. With increased stocking rate, the density of *A. frigida* population decreased linearly, while its height decreased in a step-wise fashion. The spatial distribution of *A. frigida* was mainly affected by structural factors at different scales and stocking rate. The density of *A. frigida* was more sensitive to change in stocking rate, and the patchiness distribution of *A. frigida* was more obvious with increase in scale.

**Conclusions:**

Stocking rate has a strong regulatory effect on the spatial pattern of *A. frigida* population in the desert steppe. Heavy grazing reduced the spatial heterogeneity of *A. frigida* in the desert steppe. The smaller dominant populations are unfavourable for its survival in heavy grazing condition, and affects the stability and productivity of the grassland ecosystem.

**Supplementary Information:**

The online version contains supplementary material available at 10.1186/s12870-022-03712-8.

## Introduction

Dutilleul and Legendre (1993) argued that spatial heterogeneity is extremely dependent on scale (quadrat size), and that changes in scale will lead to changes in spatial heterogeneity (or homogeneity) [1]. The causes of spatial heterogeneity are mainly due to natural disturbance, human activities and plant internal mechanisms. Grazing can increase or decrease the spatial heterogeneity of vegetation by changing the structure and spatial composition of vegetation and soil, or change the spatial pattern of soil nutrients through livestock trampling and feces emission, thereby affecting the spatial distribution of vegetation [2–4].

Long-term grazing or overgrazing is considered to be one of the important causes of degradation in arid and semi-arid grasslands, resulting in a general decline in ecosystem functioning and services [5]. An increase in stocking rate can lead to new vegetation patches on the grassland, resulting in greater fragmentation of vegetation patches [6]. Vegetation density in semi-arid grassland ecosystems is sensitive to grazing livestock [3]. Grazing can alter the spatial heterogeneity of plant populations. Changes in the number of plant populations affect the spatial pattern of plant populations and further affect the spatial heterogeneity of plant populations [7]. Lv et al. (2020) showed that the spatial distribution of *Stipa breviflora* is dependent upon the sampling unit and grazing intensity, and that the patch size of *S. breviflora* reduced with increase in sampling scale [8]. It can be seen that grazing can affect the spatial change of plant populations, but researchers mainly study the constructive species.

The desert steppe is an important type of temperate grassland vegetation in Inner Mongolia, northern of China. It is located in the transition zone between desert and grassland, and is characterized by low vegetation coverage, low precipitation and poor soil quality. In addition to climate and soil conditions, human management strategies, including grazing, affect the process of desertification [9]. Grazing time and grazing intensity affect plant species diversity and ecosystem function [10]. The study on desert steppe has important guiding significance for the sustainable development of grassland.

Shrub invasion is often considered a sign of grassland desertification. Allington and Valone (2014) showed that grazing may be the cause of the fertile island effect associated with shrubs [11]. In grasslands of Inner Mongolia, overgrazing leads to the invasion of unpalatable shrubs, such as *Caragana microphylla*, resulting in a significant decline in grassland productivity [12]. Shrubs, however, change vegetation characteristics under the canopy through seed capture, promote seed production and protect topsoil, which can promote the growth and development of other plants under the canopy and provide them with an environment to growth more than the surrounding environment (the environment after shrub seeds enter the soil is more favourable for the growth of plants) [13–15]. Therefore, some researchers believe that under some natural conditions, such as in semi-arid areas, shrubs can restore vegetation [16]. The invasion of shrubs in desert steppe in Inner Mongolia simplifies community composition at different scales and increases the spatial heterogeneity of herbaceous vegetation, so the scale effect of spatial heterogeneity of herbaceous vegetation in desert steppe mainly depends on whether the plant community is dominated by shrubs or grass [17].

**Fig. 1:**
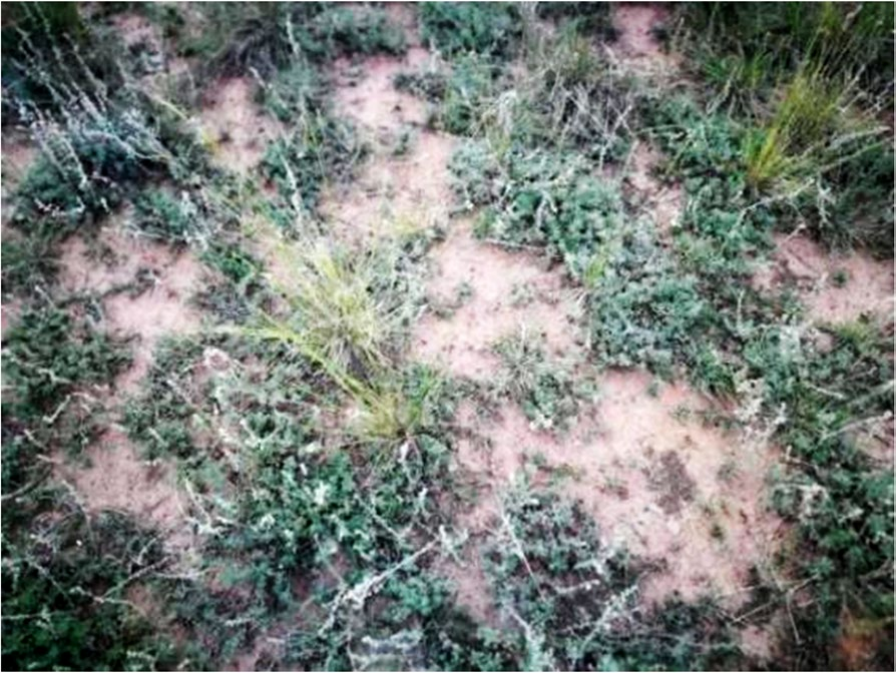
*Artemisia frigida* in the desert steppe ( taken on August 15, 2021)

*Artemisia frigida*, a small perennial semi-shrub that is a dominant species in the desert steppe of Inner Mongolia, is resistant to drought, grazing, trampling and regeneration. *A. frigida* is the main forage selected by grazing animals in winter and spring. It is the most reliable quantitative indicator plant as the structure and function of the community changes across the degradation succession gradient. In general, *A. frigida* can adapt to grazing disturbance [18]. Although the spatial heterogeneity of vegetation has been studied in different ecosystems, there have been few studies on the spatial heterogeneity of shrubs in the desert steppe. As a perennial semi-shrub and dominant species (Fig. 1, authenticated), changes in the quantitative characteristics and spatial distribution of *A. frigida* will have direct or indirect effects on the desert steppe ecosystem. A previous investigation found that the spatial distribution of dominant species *A. frigida* in Inner Mongolia desert steppe is regular [19]. Therefore, this study used geo-statistics to study the effect of stocking rate on the spatial heterogeneity of *A. frigida* in an Inner Mongolian desert steppe, and aimed to answer two questions: (1) How do the quantitative characteristics of *A. frigida* change with increasing stocking rate? (2) As stocking rate increases, how do the quantitative characteristics of *A. frigida* change on different spatial scales? In order to solve these two problems, we measured and analyzed the basic quantitative characteristics of *A. frigida* population in the long-term grazing experimental site in 2021. The study is helpful to understand the variation law of *A. frigida* plant population and provide technical and theoretical guidance for the rational and sustainable utilization of grassland.

## Methods

### Plant materials and sources

*A. frigida* samples were collected in long-term grazing experimental plots, which is located in Siziwang Banner (41° 46′ 43.6″ N, 111°53′41.7″E, elevation 1450 m) at the comprehensive experiment and demonstration center of Inner Mongolia Academy of Agriculture and Animal Husbandry Sciences, China. *A. frigida* (Fig. 1) is a wild a dominant species in desert grassland that is widely distributed in the western part of Inner Mongolia Autonomous Region, and it is not an endangered plant species. The manager of Inner Mongolia Academy of Agriculture and Animal Husbandry Sciences approved us to collect samples of *A. frigida* and identified the samples and supervised the sampling process. The area has a mid-temperate continental monsoon climate, with an average annual precipitation in the last 10 years of 220 mm. The soil texture is sandy loam [20], and the vegetation in this area is dominated by *S. breviflora*, *A. frigida* and *Cleistogenes songorica*. Vegetation is sparse, plant species composition is relatively simple, and grass coverage is low.

### Experimental design

The grazing experiment area was established in 2004, The experimental plots covered about 50 ha of natural grassland, and had been enclosed for 18 years (2004–2021). The experiment was a randomized block design. The treatments were divided into three blocks randomly. Each block contained four treatments with different stocking rates (control, CK, 0 sheep·ha^–1^·month^–1^; light grazing, LG, 0.15 sheep·ha^–1^·month^–1^; moderate grazing, MG, 0.30 sheep·ha^–1^·month^–1^; heavy grazing, HG, 0.45 sheep·ha^–1^·month^–1^), and the area of each experimental plot was 4.4 ha. The grazing numbers in CK, LG, MG and HG treatments were 0, 4, 8 and 12, respectively [21]. Grazing was conducted using Mongolian breed sheep. The daily grazing schedule was from 6 a.m. to 6 p.m., during which the sheep were free to feed. Water was provided twice a day in the morning and evening, and supplementary salt was regularly available in the form of salt bricks. The grazing period was from June to the end of November (i.e. a 6 month grazing period). During the experiment, except for stocking rate, the management measures in each grazing treatment were the same.

### Sampling and measurements

**Fig. 2:**
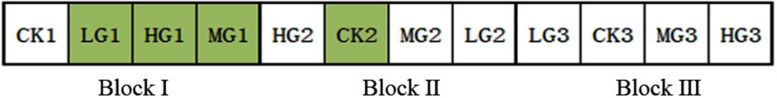
Schematic diagram of the grazing experiment treatments. Dark color treatments indicate sampling treatments

**Fig. 3:**
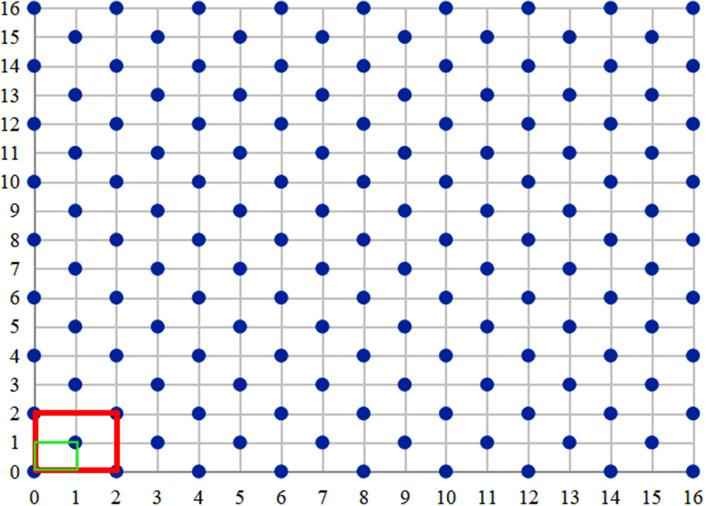
Distribution of sampling points in fixed sites. The green rectangle represents sample selection at 2.5 m × 2.5 m scale, and the red rectangle represents sample selection at 5 m × 5 m scale. The size of each experimental site was 40 m × 40 m

In order to study the spatial distribution characteristics of *A. frigida* population density and height in the desert steppe, four representative repeats with different stocking rates were selected from three replicates with the same stocking rates. As shown in Fig. 2, the CK treatment of Block II was selected to avoid the edge effect of the CK treatment in Block I. In each sample treatment with different stocking rates, a representative site (40 m × 40 m) with similar topography and located close distance to the entrance of the site was selected. A mechanical sampling method was adopted. The southwest intersection in the boundary of each sample site was set as the origin of sampling coordinates (0, 0), and a 0.5 m × 0.5 m quadrat was set every 5 m, and the farthest coordinate from the origin was (16, 16). Nine quadrats were taken from even lines and eight from odd lines for sampling. As shown in Fig. 3, where each blue circle represents a 50 cm × 50 cm quadrat, there were 145 quadrats in each site. Sampling was conducted on August 15, 2021.

### Statistical analyses

The basic quantitative characteristics of *A. frigida* analyzed were density (cluster / m^2^) and height (cm), and the sample sizes were 256 and 64 at scales of 2.5 m × 2.5 m and 5 m × 5 m, respectively. In order to compare the changes in the density and height of *A. frigida* at different scales and stocking rates, box plots were used for comparative analysis, and graphs were plotted using Sigmaplot 14.0 (Systat Software, 2011). The box diagram can represent the concentration and dispersion of the sample, and the distance between the two ends of the box diagram represents the overall distribution of the sample data.

To test whether the density and height of *A. frigida* have significant differences at different scales and stocking rates, the density and height data were square root transformed to approximate a normal distribution, and the normal distribution test with known overall mean and variance was used. The mean *µ* and variance σ^2^ of each CK treatment on two scales were taken as the overall mean and variance, and the mean and variance of other treatments were taken as the mean and variance of the sample. The mean and variance obtained by CK treatment and other treatments were statistically tested.

The skewness, kurtosis and confidence interval of the sample data distribution were calculated. All skewness and kurtosis were included in the confidence interval, and the sample data were normally distributed. The calculations were performed in Excel 2010 (Microsoft Inc.). Therefore, we conclude that the sample data follow a normal distribution.

Furthermore, geo-statistical methods were used to analyze the density and height of *A. frigida* populations within 2.5 × 2.5 m and 5 × 5 m sample sites. Geological statistical methods can evaluate spatial data structure and auto-correlation by optimal estimation of semi-variance model parameters [22–24].

The semi-variance model parameters mainly include *C*_0_, *C*_0_ + *C*, *C/*(*C*_0_ + *C*), *A*_0_, *D*_0_. Nugget variance (*C*_0_) reflects the randomness of spatial variation, which is caused by random factors. *C*_0_ + *C* represents the semi-variance maxima at different sampling intervals and reflects the spatial variation caused by structural variation factors and random variation factors. *C/*(*C*_0_ + *C*) is the proportion of spatial variability of structural factors in total variability, reflecting the extent to which the spatial heterogeneity of structural components (i.e., spatial variability caused by structural factors such as topography, soil parent material, and climate) accounts for total spatial heterogeneity [25]. The range parameter *A*_0_ is used to indicate the spatial correlation range of variables. Fractal dimension *D*_0_ is an important dimensionless index used to compare the spatial dependence between different variables or spatial scales. We used the residual sum of squares (RSS) of the least squares method to select linear, spherical, exponential, and Gaussian models [26]. Different models have different spatial auto-correlation ranges, and the spatial auto-correlation ranges of linear, exponential, spherical and Gaussian models are *A*_0_, 3*A*_0_, *A*_0_ and $\sqrt{3}$  *A*_0_.

The kriging method was used for spatial interpolation, and the spatial distribution map of *A. frigida* was drawn according to the semi-variance function [27]. Geo-statistical analysis was performed using GS + software (Version 9, Gamma Design software, 2014).

## Results

### Quantitative characteristics of *Artemisia frigida*

**Fig. 4:**
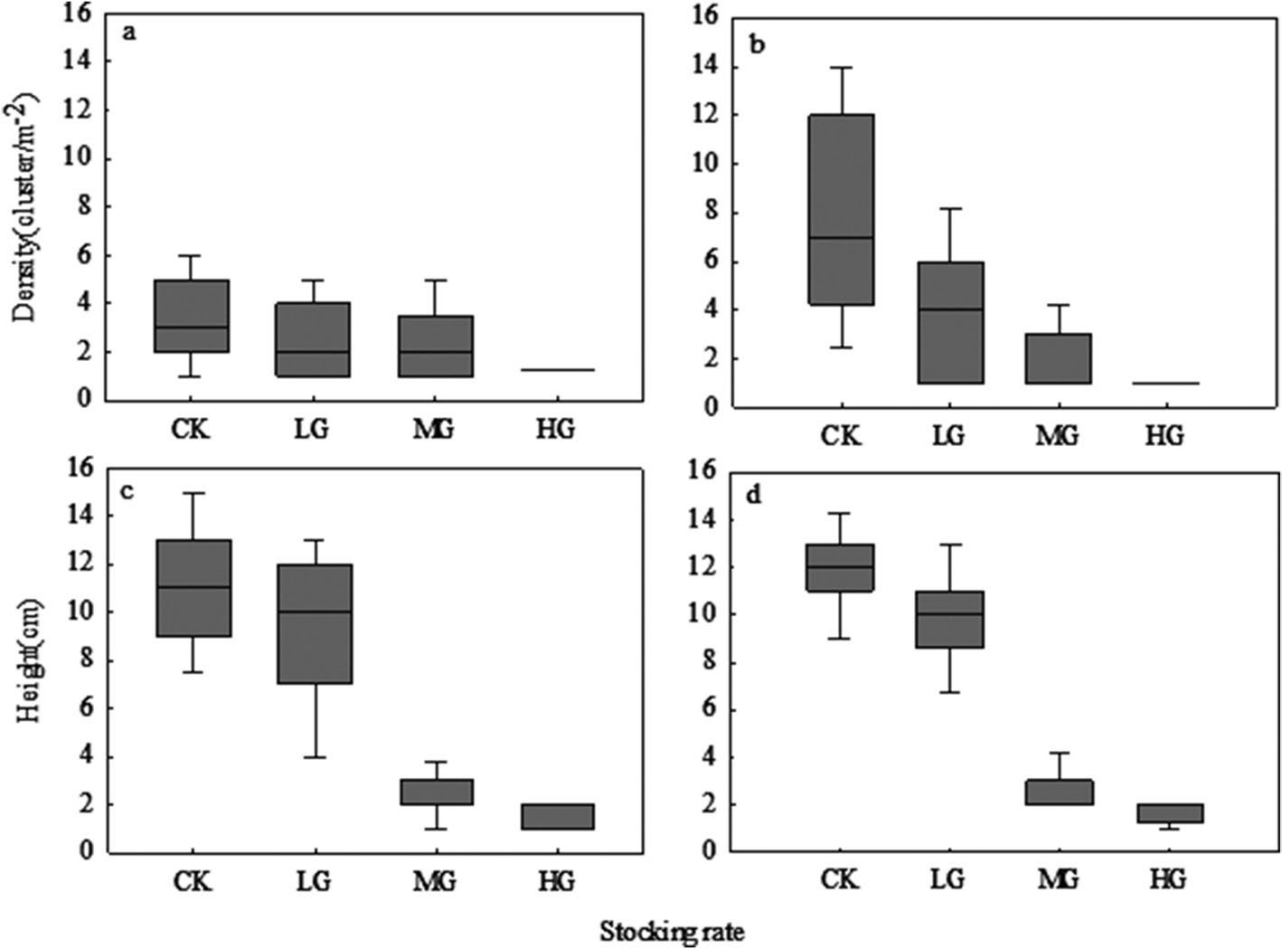
Changes in density and height of *Artemisia frigida* at different scales and stocking rates. **a** *Artemisia frigida* density at 2.5 m × 2.5 m; (**b**) *Artemisia frigida* density at 5 m × 5 m; (**c**) *Artemisia frigida* height at 2.5 m × 2.5 m; (**d**) *Artemisia frigida* height at 5 m × 5 m. CK, control treatment; LG, light grazing; MG, moderate grazing; HG, heavy grazing

Hypothesis testing confirmed significant differences in the density and height of *A. frigida* at different scales and stocking rates (*P* < 0.05). Compared with 2.5 m × 2.5 m scale, the density of *A. frigida* at the scale of 5 m × 5 m had a large distribution range (Fig. 4a and b). Among the height changes of *A. frigida* at different scales, the height variation of *A. frigida* was largest in LG treatment at 2.5 m × 2.5 m scale (Fig. 4c and d). Overall, as scale increased, the median density of *A. frigida* (the solid line in each boxplot) showed a clear increase, while the median height of *A. frigida* increases but not obvious. As stocking rate increased, the density of *A. frigida* at both scales showed a linear downward trend, while the height of *A. frigida* showed a stepwise downward trend.

### The effects of stocking rate on *Artemisia frigida* spatial heterogeneity

**Fig. 5:**
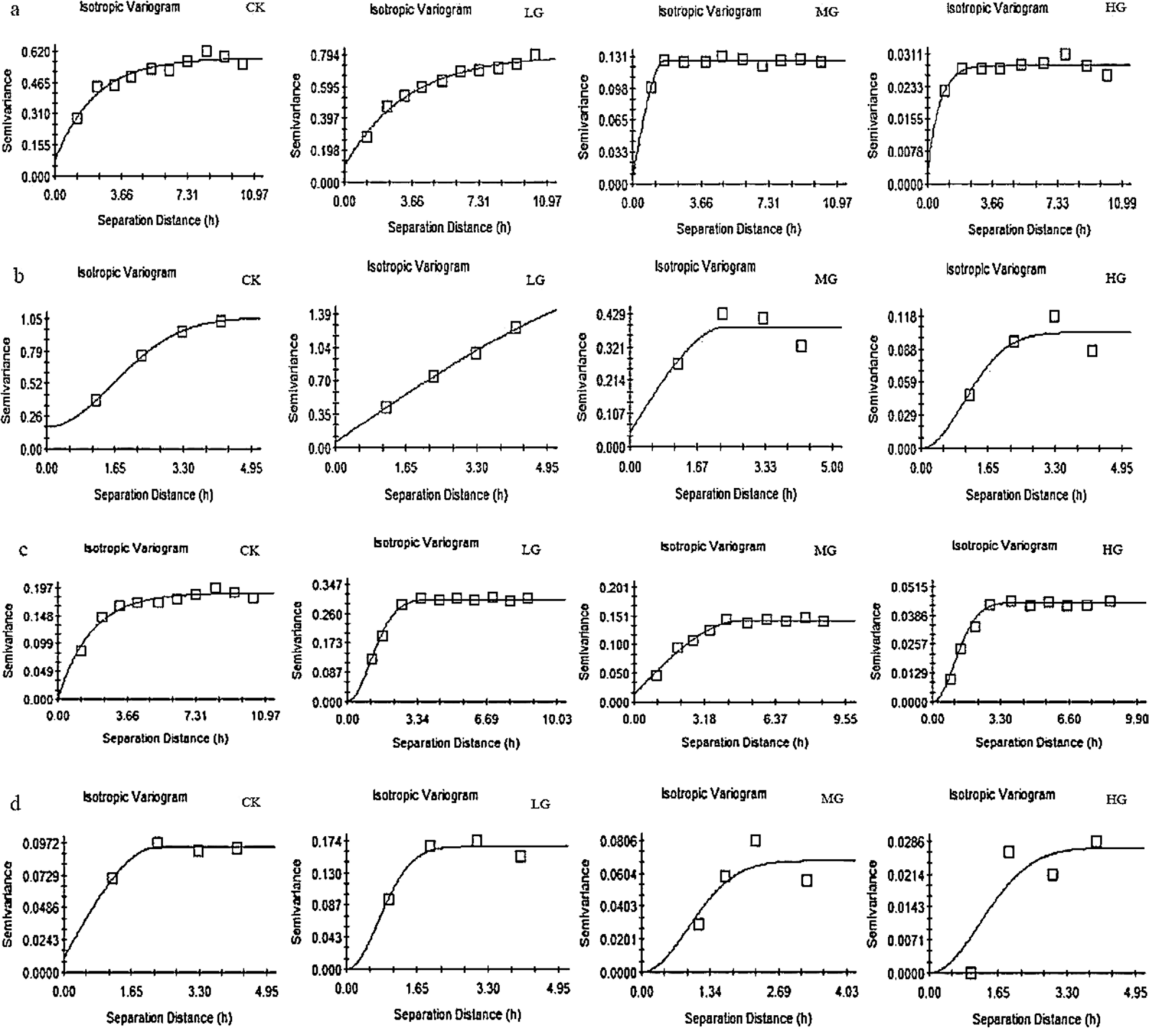
Semi-variograms of *Artemisia frigida* spatial distribution at different stocking rates. **a** *Artemisia frigida* density at 2.5 m × 2.5 m; (**b**) *Artemisia frigida* density at 5 m × 5 m; (**c**) *Artemisia frigida* height at 2.5 m × 2.5 m; (**d**) *Artemisia frigida* height at 5 m × 5 m. CK, control treatment; LG, light grazing; MG, moderate grazing; HG, heavy grazing

Variation function analysis of *A. frigida* density and height at different scales and stocking rates showed that different mathematical models are applicable at different scales and stocking rates. Table 1 shows that the optimal models for the density of *A. frigida* at 2.5 m × 2.5 m scale were Exponential and Spherical models, while the optimal models for the density of *A. frigida* at 5 m × 5 m scale were Gaussian and Spherical models. At 2.5 m × 2.5 m scale, the optimal models for the height were Exponential, Gaussian and Spherical models, and at 5 m × 5 m scale the optimal models were Gaussian and Spherical models. As shown in Fig. 5, the value of semi-variance functions gradually increased with increasing spatial sampling separation distance, but tended to be stable after a certain separation distance had been reached. The model fitting residuals at different scales and stocking rates were small.

**Table 1:** Relevant indicators of curve-fitted semi-variograms at different scales in different stocking rate treatments

Quantitative characteristics	Scale	Stocking rate	Model parameter		Semi-variogram			Patch parameters		
			Model	RSS	*C*_0_	*C*_0_ + *C*	*C/*(*C*_0_ + *C*)(%)	*A*_0_	Auto-corr. Ranges(m)	*D*_0_
Density	2.5 m × 2.5 m(a)	CK	Exponential	6.168 × 10^–3^	0.077	0.587	86.882	5.575	16.725	1.847
		LG	Exponential	3.700 × 10^–3^	0.101	0.792	87.247	8.525	25.569	1.783
		MG	Spherical	5.597 × 10^–5^	0.007	0.127	94.488	4.300	4.300	1.966
		HG	Exponential	1.355 × 10^–5^	0.003	0.028	89.286	1.700	5.100	1.961
	5 m × 5 m(b)	CK	Gaussian	7.012 × 10^–5^	0.176	1.057	83.349	11.300	19.572	1.603
		LG	Spherical	7.504 × 10^–4^	0.057	1.759	96.760	41.900	41.900	1.574
		MG	Spherical	6.638 × 10^–3^	0.047	0.387	87.855	12.050	12.050	1.898
		HG	Gaussian	4.987 × 10^–4^	0.000	0.103	99.903	7.350	12.730	1.713
Height	2.5 m × 2.5 m(c)	CK	Exponential	4.177 × 10^–4^	0.001	0.188	99.468	4.375	13.125	1.844
		LG	Gaussian	6.064 × 10^–3^	0.002	0.302	99.338	4.000	6.928	1.829
		MG	Spherical	5.685 × 10^–3^	0.014	0.142	90.141	11.975	11.975	1.800
		HG	Gaussian	6.324 × 10^–4^	0.001	0.044	97.727	3.800	6.582	1.968
	5 m × 5 m(d)	CK	Spherical	1.849 × 10^–5^	0.011	0.094	88.298	11.300	11.300	1.892
		LG	Gaussian	3.045 × 10^–4^	0.001	0.166	99.398	5.300	9.180	1.819
		MG	Gaussian	4.267 × 10^–4^	0.001	0.068	98.529	6.450	11.171	1.648
		HG	Gaussian	1.339 × 10^–4^	0.000	0.027	99.630	8.550	14.809	1.967

It can be seen from the fitted semi-variance functions (Table 1) at different scales and stocking rates that the structural ratios *C/*(*C*_0_ + *C*) were greater than 85%, indicating that the spatial correlation of *A. frigida* changed little at different scales and stocking rates, and that *A. frigida* spatial variation is mainly affected by structural factors.

Based on analysis of semi-variance functions, the patch parameters were further studied (Table 1). At 2.5 m × 2.5 m scale, the fractal dimension *D*_*0*_ represents the proportion of structural spatial distribution factors in the maximum spatial variation, and it is obvious that the *D*_*0*_ value in LG treatment was the smallest. At the scale of 5 m × 5 m, the *D*_*0*_ value of the LG treatment was the smallest. At both scales, the spatial heterogeneity of *A. frigida* density in the LG treatment was the most obvious and the patches were large. By comparing the patch parameters of *A. frigida* density at each scale, it can be seen that as spatial scale increased, the patch density of *A. frigida* increased and spatial heterogeneity was enhanced.

Analysis of the patch parameters for *A. frigida* (Table 1) showed that at the scale of 2.5 m × 2.5 m, the maximum spatial auto-correlation range of the two scales was in the CK treatment, and spatial heterogeneity was the highest in the MG treatment. However, from the spatial auto-correlation scale *A*_*0*_, it can be seen that the patchiness of *A. frigida* height in the CK treatment was larger at 2.5 m × 2.5 m, while it was larger at 5 m × 5 m in the MG treatment, indicating that with the increase of spatial scale, the patchiness increased from CK to MG treatment.

Overall, compared with the height of *A. frigida*, density of *A. frigida* had a stronger response to stocking rate. For both density and height of *A. frigida* at different scales, the fractal dimension *D*_*0*_ values in the HG treatment was high, which was mainly because the number of *A. frigida* decreased sharply in the HG treatment, with only a few scattered plants distributed accompanied by extensive bare land. Heavy grazing thus reduced the spatial heterogeneity of *A. frigida*.

### The spatial distribution of *Artemisia frigida* population at different stocking rates

The two dimensional spatial distribution patterns of *A. frigida* can reflect its degradation succession stage under grazing pressure. The spatial distribution of *A. frigida* in different grazing treatments was plotted using the kriging method for interpolation to obtain a spatial distribution map showing the heterogeneity and complexity of the spatial distribution of *A. frigida*, as well as the distribution characteristics of patchiness, gradient and mosaic [28].

**Fig. 6:**
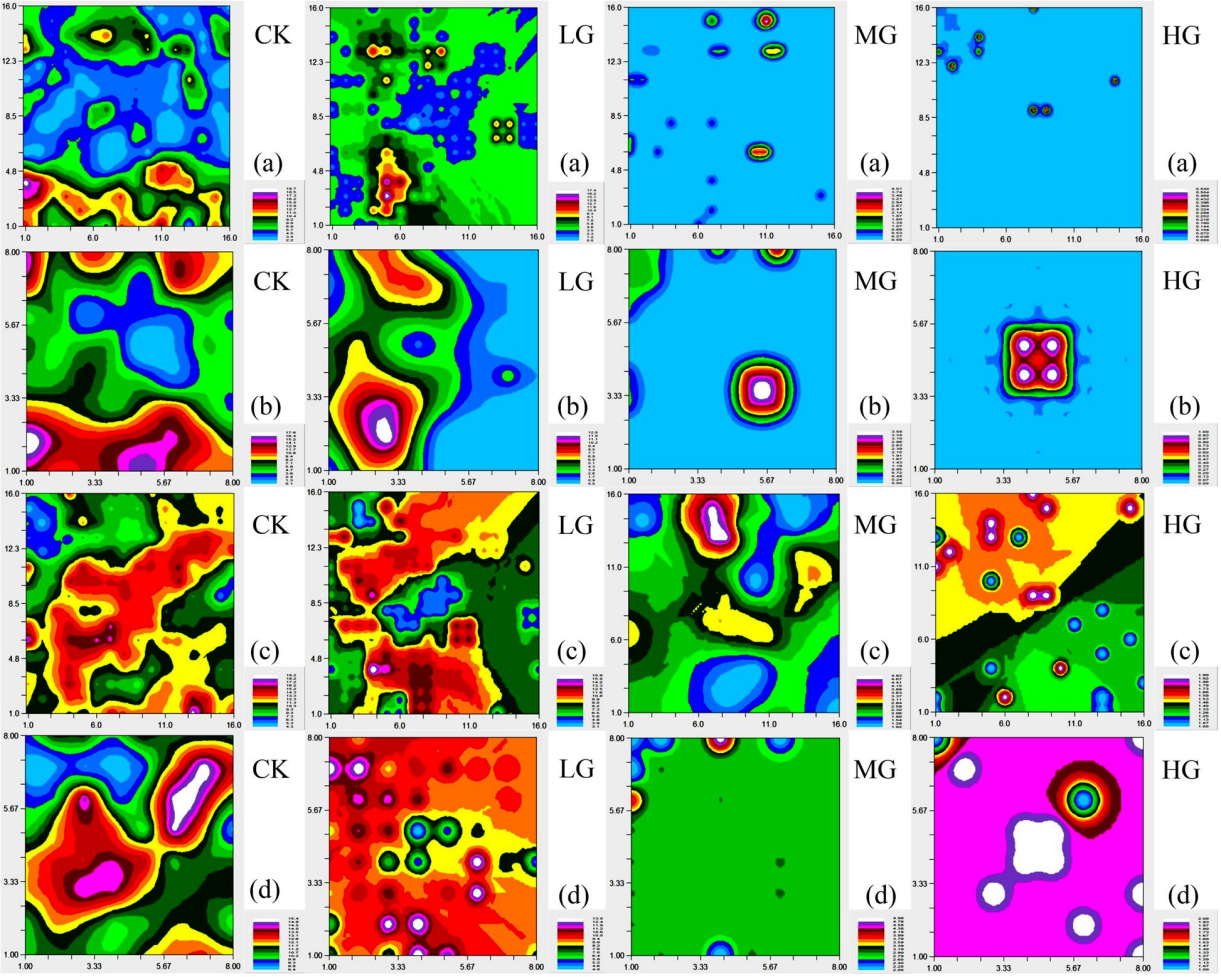
The two dimensional spatial pattern map of *Artemisia frigida* at different stocking rates. **a** *Artemisia frigida* density at 2.5 m × 2.5 m; (**b**) *Artemisia frigida* density at 5 m × 5 m; (**c**) *Artemisia frigida* height at 2.5 m × 2.5 m; (**d**) *Artemisia frigida* height at 5 m × 5 m. CK, control treatment; LG, light grazing; MG, moderate grazing; HG, heavy grazing. Figures show the quantitative characteristics of *Artemisia frigida*. The color of the figure from white to blue indicates the value from big to small. The first half is the north, the second half is the south, the left half is the west, and the right half is the east

The spatial distribution of *A. frigida* density at different scales is shown in Fig. 6a and b. At the scale of 2.5 m × 2.5 m, the CK treatment showed two kinds of zonal distribution. The density of *A. frigida* with zonal distribution in the north was smaller than that in the south. The LG treatment showed a patchy distribution, and the density of *A. frigida* increased moving from the center to the surrounding treatments. The density distributions of *A. frigida* in the MG and HG treatments were lower than in the CK and LG treatments, and showed a decentralized patchy distribution, but there were more patches in the MG treatment than in the HG treatment.

At the scale of 5 m × 5 m, in the CK treatment, the density spatial distribution of *A. frigida* showed two kinds of zonal distribution, the density in the south was large and the density in the north was small. In the LG treatment, it can be seen that *A. frigida* was mainly concentrated in the west of the sample site, with a high density of *A. frigida* in the southwest corner and a low density in the northwest corner was small, indicating two kinds of zonal distribution. In the MG treatment, the density of *A. frigida* was concentrated in the center of the sample site and presents a rectangular pattern, with density decreasing from the center of the sample site to the surrounding areas. In the HG treatment, *A. frigida* only showed a circular distribution in the southeast corner. In this circular area, the density of *A. frigida* gradually decreased from the center to the periphery. As stocking rate increased, the distribution of *A. frigida* density at both spatial scales tends to be singleness.

Comparing the two scales, it can be seen that the distribution of *A. frigida* at the scale of 2.5 m × 2.5 m is relatively dispersed. As spatial scale increased, the density of *A. frigida* increased, the patchiness degree decreases, and the patchiness size increases, showing a zonal distribution, and the spatial aggregation of *A. frigida* increases.

The spatial distribution of *A. frigida* height at different scales is shown in Fig. 6c and d. At the scale of 2.5 m × 2.5 m, the CK treatment showed a larger patch distribution, and the height of *A. frigida* was mainly concentrated in the center of the site, while *A. frigida* plants around the site edges tended to be smaller in height. The LG treatment showed a mixture of large and small patches. The height value was mostly concentrated in the center of the northwest and south of the site, and the height value of *A. frigida* in the east was lower. The height of *A. frigida* in MG treatment had a zonal distribution, with a larger value in the center of the northern part of the site, and a smaller in the center of the south of the site. The height of *A. frigida* was more uniform in other locations in the MG treatment. The HG treatment showed a small patch distribution. The height of *A. frigida* decreased from the southeast corner to the northwest corner of the HG treatment.

At the scale of 5 m × 5 m, the CK treatment showed a zonal distribution, and the height value decreased from the center of the plot to the periphery, and the height value of *A. frigida* was larger in the northeast and southwest corners. The height of *A. frigida* in the LG treatment had a patchy distribution, with a smaller height value in the center of the LG treatment and a larger value towards the plot edges. The MG treatment showed a semi-circular patch distribution, and the number of *A. frigida* plants was reduced. Height had a notable distribution in a small part at the center of the north of the site, and had a relatively uniform distribution in other locations. The HG treatment, showed a large patch distribution, with higher height value only the center and a decline from the northeast corner towards the center. As stocking rate increased, the height of *A. frigida* decreased. Although the spatial distribution of *A. frigida* height at the scale of 2.5 m × 2.5 m tended to be simple, the degree of spatial heterogeneity was weak, whereas the spatial heterogeneity of *A. frigida* height at the scale of 5 m × 5 m was obvious.

Combined with the structure ratio, it can be seen that the structure ratio decreased as stocking rate increased, while the spatial auto-correlation weakened (except in the LG treatment), indicating that increase in stocking rate and scale tends to simplify the spatial distribution of *A. frigida* height.

## Discussion

The characteristics of grazed grassland plant communities are closely related to stocking rate. *Artemisia frigida*, a dominant species in desert grassland plant communities, is no exception. When climate and soil factors are held constant, grazing intensity will become the dominant factor, controlling and influencing the characteristics of plant communities [29].

Most studies have shown that grazing can lead to changes in vegetation, such as decrease in the frequency of palatable plants, and increases in shrub density and the proportion of shrubs in plant community [30, 31]. However, under long-term moderate grazing, the relative importance of shrubs increased, and this effect gradually weakened under extreme grazing levels [32]. In this study, the density and height of *A. frigida* showed a downward trend with increasing stocking rate, but the decline in density was linear while height declined in a step-wise pattern. Grazing affects *A. frigida* density and grazing intensity affects *A. frigida* height. This indicates that although *A. frigida* is a grazing-resistant plant, its plant population characteristics will decrease with the increase of stocking rate. *A. frigida* is mainly asexual reproduction, grazing disturbance may change the reproductive mode of *A. frigida* [33–35].

Studies have also shown that under long-term heavy grazing, the decline in plant height is a morphological adaptation to herbivory and the most important ' grazing avoidance ' strategy of plants [36]. The experimental plot of this study is a desert steppe in Inner Mongolia, where temperature and precipitation factors also limit the growth and development of plants. The resistance and resilience of plants to grazing disturbance in *Stipa breviflora* desert steppe mainly depend on precipitation conditions, and moderate disturbance is beneficial to their growth [37]. Climatic factors may also be one of the reasons for the decline in density of *A. frigida* populations in the desert steppe.

Compared with herbaceous plants, shrubs have weaker spatial auto-correlation, a more obvious fragmented distribution and higher spatial heterogeneity, but increased grazing intensity will reduce the spatial heterogeneity of both shrubs and herbs [17]. In this study, from the perspective of the spatial distribution of *A. frigida* density, the patchiness in LG treatment was the most obvious, and the fractal dimension *D*_*0*_ in the MG treatment was the largest, indicating that the distribution of *A. frigida* density in the MG treatment was uniform. From the spatial distribution of *A. frigida* height, the spatial heterogeneity increased with increasing stocking rate, and the spatial heterogeneity was the largest in the MG treatment. The spatial distribution of the density and height of *A. frigida* was relatively uniform in the CK and HG treatments. Thus, the density of *A. frigida* was more sensitive to stocking rate than height. Under moderate grazing conditions, the selective grazing and trampling of livestock were relatively weak, which would promote the formation of adventitious roots and the ability of *A. frigida* to sprout tillers. After the apical dominance is destroyed, the tip and aging tissues of *A. frigida* plants are removed, and the semi-stolon branches generate adventitious roots for clonal growth to absorb nutrients and obtain resources [38]. This conclusion supports the research results of this study.

Livestock excrement provides good nutrients for the growth and development of *A. frigida*. The developed roots of *A. frigida* will thus be able to absorb a lot of nutrients, indicating that grazing creates a relatively stable environment for *A. frigida*. Due to the allelopathy of *A. frigida*, the stems and leaves of *A. frigida* often release volatile substances that can inhibit animal feeding, seed germination, seedling growth and reproduction of other forages, and enhance the survival competitiveness of *A. frigida*, enabling it to occupy a dominant position in the plant community [39]. These characteristics provide a solid foundation for building a stable soil ecological community [40]. Since the number of *A. frigida* plants decreased sharply with the increase of stocking rate, the fractal dimension *D*_*0*_ of HG treatment was large, indicating that *A. frigida* was sporadically distributed. The nutrient supply rate to *A. frigida* itself could not be maintained due to the influence of livestock feeding, so the number of *A. frigida* plants decreased significantly, the physical and chemical properties of soil were destroyed, and the living environment of microorganisms deteriorated, which was unfavourable for the growth of *A. frigida* [41, 42].

Spatial scale will affect the spatial heterogeneity of the *A. frigida* population, further highlighting the important role of spatial scale in the spatial pattern and heterogeneity of vegetation distribution [43]. In this study, compared with the smaller scale, the spatial distribution of *A. frigida* at large scale was more sensitive and had higher spatial heterogeneity. This is similar to the results of He and Zhao's study of riparian vegetation that showed significant spatial variability at a large scale [44]. This may be due to the effect of environmental factors influencing the vegetation spatial heterogeneity of the *A. frigida* population at large scale [45]. Studies have shown that the spatial heterogeneity of desert steppe vegetation is determined by spatial scale changes. As spatial scale increases, spatial heterogeneity of vegetation is likely to be influenced by the spatial heterogeneity of terrain characteristics and soil properties [17]. However, the reason why vegetation spatial heterogeneity changes with increasing scale is not clear. Therefore, in order to better understand the impact of shrub invasion on multi-scale vegetation spatial heterogeneity, future research should strive to explore the internal mechanisms driving vegetation spatial heterogeneity at different scales.

## Conclusions

The conclusion of this study is that heavy grazing inhibits the spatial heterogeneity of *Artemisia frigida*. The increase of spatial scale will enhance the spatial heterogeneity of *A. frigida*, but with the increase of stocking rate, the spatial heterogeneity of *A. frigida* tends to be simplified, indicating that the spatial pattern of *A. frigida* in desert steppe plant population is different at different scales. Grazing rate has a strong regulatory effect on the spatial pattern of *A. frigida* population in desert steppe, affecting the stability and productivity of grassland ecosystem.

## Supplementary Information

**Additional file 1.** Data.

**Additional file 2.** Highlights.

**Additional file 3.** Plant identification proof.

**Additional file 4.** Sample license.

**Additional file 5.** Affiliations.

## Data Availability

The data used to support the findings of this study are available from the corresponding author upon request.

## Abbreviations

CK: No grazing
LG: Light grazing
MG: Moderate grazing
HG: Heavy grazing

## References

[1] Dutilleul P, Legendre P. Spatial heterogeneity against heteroscedasticity: an ecological paradigm versus a statistical concept. Oikos 1993: 152–171. 10.2307/3545210.

[2] Augustine D J, McNaughton S J. Interactive effects of ungulate herbivores, soil fertility, and variable rainfall on ecosystem processes in a semi-arid savanna. Ecosystems 2006; 9: 1242–1256.https://link.springer.com/article/10.1007/s10021-005-0020-y.

[3] Knapp A K, Fay P A, Blair J M, Collins S L, Smith M D, Carlisle J D, et al. Rainfall variability, carbon cycling, and plant species diversity in a mesic grassland. science 2002; 298: 2202–2205. https://www.science.org/doi/10.1126/science.1076347. 10.1126/science.1076347 12481139

[4] Schlesinger WH, Raikes JA, Hartley AE, Cross AF (1996). On the spatial pattern of soil nutrients in desert ecosystems: ecological archives E077–002. Ecology.

[5] Bai Y, Wu J, Clark CM, Pan Q, Zhang L, Chen S (2012). Grazing alters ecosystem functioning and C: N: P stoichiometry of grasslands along a regional precipitation gradient. J Appl Ecol.

[6] Bisigato AJ, Bertiller MB (1997). Grazing effects on patchy dryland vegetation in northern Patagonia. J Arid Environ.

[7] Adler P, Raff D, Lauenroth W. The effect of grazing on the spatial heterogeneity of vegetation. Oecologia 2001; 128: 465–479. https://link.springer.com/article/10.1007/s004420100737. 10.1007/s004420100737 28547391

[8] Lv S, Yan B, Wang Z, Wang Z, Song X, Zhao M (2020). Dominant species' dominant role and spatial stability are enhanced with increasing stocking rate. Sci Total Environ.

[9] Zhang R, Wang Z, Han G, Schellenberg MP, Wu Q, Gu C (2018). Grazing induced changes in plant diversity is a critical factor controlling grassland productivity in the Desert Steppe, Northern China. Agr Ecosyst Environ.

[10] Milchunas D G, Sala O E, Lauenroth W K. A generalized model of the effects of grazing by large herbivores on grassland community structure. The American Naturalist 1988; 132: 87–106. https://sci-hub.se/10.2307/2461755.

[11] Allington G R, Valone T J. Islands of fertility: a byproduct of grazing? Ecosystems 2014; 17: 127–141. https://linkspringer.53yu.com/article/10.1007/s10021-013-9711-y.

[12] Yan Y, Xu D, Xu X, Wang D, Wang X, Cai Y, et al. Shrub patches capture tumble plants: potential evidence for a self-reinforcing pattern in a semiarid shrub encroached grassland. Plant and Soil 2019; 442: 311–321. https://link.springer.com/article/10.1007/s11104-019-04189-5.

[13] Abdulahi M M, Ebro A, Nigatu L. Impact of woody plants species on soil physio-chemical properties along grazing gradients in rangelands of Eastern Ethiopia. Tropical and Subtropical Agroecosystems 2016; 19. https://www.revista.ccba.uady.mx/ojs/index.php/TSA/article/view-/2254.

[14] Erfanzadeh R, Shahbazian R, Zali H. Role of plant patches in preserving flora from the soil seed bank in an overgrazed high-mountain habitat in northern Iran. Journal of Agricultural Science and Technology 2014; 16: 229–238. https://jast.modares.ac.ir/article-23-1688-en.html.

[15] García-Sánchez R, Camargo-Ricalde S L, García-Moya E, Luna-Cavazos M, Romero-Manzanares A, Manuel Montaño N. Prosopis laevigata and Mimosa biuncifera (Leguminosae), jointly influence plant diversity and soil fertility of a Mexican semiarid ecosystem. Revista de Biología Tropical 2012; 60: 87–103. https://www.scielo.sa.cr/scielo.php?script=sci_artt-ext&pid=S0034-77442012000100006. 22458211

[16] Ren H, Yang L, Liu N (2008). Nurse plant theory and its application in ecological restoration in lower subtropics of China. Prog Nat Sci.

[17] Zuo X, Mao W, Qu H, Chen M, Zhao S, Liu L (2021). Scale effects on spatial heterogeneity of herbaceous vegetation in desert steppe depend on plant community type. Ecol Ind.

[18] Müller I, Schmid B, Weiner J (2000). The effect of nutrient availability on biomass allocation patterns in 27 species of herbaceous plants. Perspectives in plant ecology, evolution and systematics.

[19] Liu Z, Li Z, Dong M, Nijs I, Bogaert J, El-Bana M I. Small-scale spatial associations between Artemisia frigida and Potentilla acaulis at different intensities of sheep grazing[J]. Applied Vegetation Science,2007,10(1), 139–148. https://www.jstor.org/stable/4620508.

[20] Wang Z, Jiao S, Han G, Zhao M, Willms WD, Hao X (2011). Impact of stocking rate and rainfall on sheep performance in a desert steppe. Rangel Ecol Manage.

[21] Wang ZW, Jiao SY, Han GD, Zhao ML, Willms WD, Hao XY, Wang JA, Din HJ, Havstad KM (2011). Impact of stocking rate and rainfall on sheep performance in a desert steppe[J]. Rangel Ecol Manage.

[22] Makarian H, Mohassel MR, Bannayan M, Nassiri M (2007). Soil seed bank and seedling populations of Hordeum murinum and Cardaria draba in saffron fields. Agr Ecosyst Environ.

[23] Yavitt J, Harms K, Garcia M, Wright S, He F, Mirabello M. Spatial heterogeneity of soil chemical properties in a lowland tropical moist forest, Panama. Soil Research 2009; 47: 674–687. https://sci-hub.se/10.1071/SR08258.

[24] Zhou Z, Sun O J, Luo Z, Jin H, Chen Q, Han X. Variation in small-scale spatial heterogeneity of soil properties and vegetation with different land use in semiarid grassland ecosystem. Plant and Soil 2008; 310: 103–112. https://link.springer.com/article/10.1007/s11104-008-9633-1.

[25] Lv S, Yan B, Wang Z, Han G, Kang S (2019). Grazing intensity enhances spatial aggregation of dominant species in a desert steppe. Ecol Evol.

[26] Augustine DJ, Frank DA (2001). Effects of migratory grazers on spatial heterogeneity of soil nitrogen properties in a grassland ecosystem. Ecology.

[27] Matheron G (1963). Principles of geostatistics Economic geology.

[28] Matheron G. A simple substitute for conditional expectation: the disjunctive kriging. Advanced geostatistics in the mining industry. Springer, 1976, pp. 221–236. https://link.springer.com/ch-apter/10.1007/978-94-010-1470-0_14?noAccess=true.

[29] Shan Y, Chen D, Guan X, Zheng S, Chen H, Wang M (2011). Seasonally dependent impacts of grazing on soil nitrogen mineralization and linkages to ecosystem functioning in Inner Mongolia grassland. Soil Biol Biochem.

[30] Campanella M, Bisigato A J, Rostagno C M. Plant production along a grazing gradient in a semiarid Patagonian rangeland, Argentina. Plant ecology 2016; 217: 1553–1562. https://sci-hub.wf/doi:10.1007/s11258-016-0668-8.

[31] Chartier M, Rostagno C, Pazos G (2011). Effects of soil degradation on infiltration rates in grazed semiarid rangelands of northeastern Patagonia. Argentina Journal of Arid Environments.

[32] Cai Y, Yan Y, Xu D, Xu X, Wang C, Wang X (2020). The fertile island effect collapses under extreme overgrazing: evidence from a shrub-encroached grassland. Plant Soil.

[33] Li XF, Wang J, Huang D, Wang LX, Wang K. Allelopathic potential of Artemisia frigida and successional changes of plant communities in the northern China steppe. Plant and Soil 2011; 341: 383–398. https://sci-hub.se/10.1007/s11104-010-0652-3.

[34] Zhang R, Zuo Z, Gao P, Hou P, Wen G, Gao Y. Allelopathic effects of VOCs of Artemisia frigida Willd. on the regeneration of pasture grasses in Inner Mongolia. Journal of arid environments 2012; 87: 212–218. 10.1016/j.jaridenv.2012.04.008.

[35] Ma R, Wang M, Zhu X, Lu X, Sun K. Allelopathy and chemical constituents of Ligularia virgaurea volatile. Ying Yong Sheng tai xue bao= The Journal of Applied Ecology 2005; 16: 1826–1829. https://europepmc.org/article/med/16422498. 16422498

[36] Lemaire G, Chapman D. Tissue flows in grazed plant communities. Cab international. 1996. https://hal.inrae.fr/hal-02836461.

[37] Ye R, Liu G, Chang H, Shan Y, Mu L, Wen C (2020). Response of plant traits of Stipa breviflora to grazing intensity and fluctuation in annual precipitation in a desert steppe, northern China. Global Ecology and Conservation.

[38] Li X F, Wang J, Huang D, Wang L X, Wang K. Allelopathic potential of Artemisia frigida and successional changes of plant communities in the northern China steppe. Plant and Soil 2011; 341: 383–398. https://linkspringer.53yu.com/article/10.1007/s11104-010-0652-3.

[39] Hassan S, Aidy I, Bastawisi A. Weed management in rice using allelopathic rice varities in Egypt. 1996. International Workshop on Allelopathy in Rice, International Rice Research Institute, Los Banos, Philippines. https://www.researchgate.net/publication/248936648.

[40] Zhang R, Zhang W, Zuo Z, Li R, Wu J, Gao Y. Inhibition effects of volatile organic compounds from Artemisia frigida Willd. on the pasture grass intake by lambs. Small Ruminant Research 2014; 121: 248–254. 10.1016/j.smallrumres.2014.06.001.

[41] Inderjit. Plant phenolics in allelopathy. The Botanical Review 1996: 186–202. https://www.jstor.org/stable/4354269.

[42] Sunohara Y, Baba Y, Matsuyama S, Fujimura K, Matsumoto H. Screening and identification of phytotoxic volatile compounds in medicinal plants and characterizations of a selected compound, eucarvone. Protoplasma 2015; 252: 1047–1059. https://sci-hub.se/10.1007/s00709-014-0739-4. 10.1007/s00709-014-0739-4 25534256

[43] Liu H, Zhang M, Lin Z, Xu X (2018). Spatial heterogeneity of the relationship between vegetation dynamics and climate change and their driving forces at multiple time scales in Southwest China. Agric For Meteorol.

[44] He Z, Zhao W (2006). Characterizing the spatial structures of riparian plant communities in the lower reaches of the Heihe River in China using geostatistical techniques. Ecol Res.

[45] Schooley RL (2006). Spatial heterogeneity and characteristic scales of species-habitat relationships. Bioscience.

