# Supplementary material for: Heavy grazing reduced the spatial heterogeneity of Artemisia frigida in desert steppe
Source: BMC Plant Biol. 2022 Jul 13;22:337. doi: 10.1186/s12870-022-03712-8 (PMC9281028; doi:10.1186/s12870-022-03712-8)
Supplement: Supplementary file 2 — Additional file 2. Highlights. [file 12870_2022_3712_MOESM2_ESM.docx]

Highlights:

- The grazing tolerance of *Artemisia frigida* decreased with the increase of stocking rate.
- An increase in spatial scale enhanced the spatial heterogeneity of *Artemisia frigida*.
- Spatial heterogeneity of *Artemisia frigida* population was inhibited under heavy grazing.
