# Supplementary figures and images for: Heavy grazing reduced the spatial heterogeneity of Artemisia frigida in desert steppe
Source: BMC Plant Biol. 2022 Jul 13;22:337. doi: 10.1186/s12870-022-03712-8 (PMC9281028; doi:10.1186/s12870-022-03712-8)

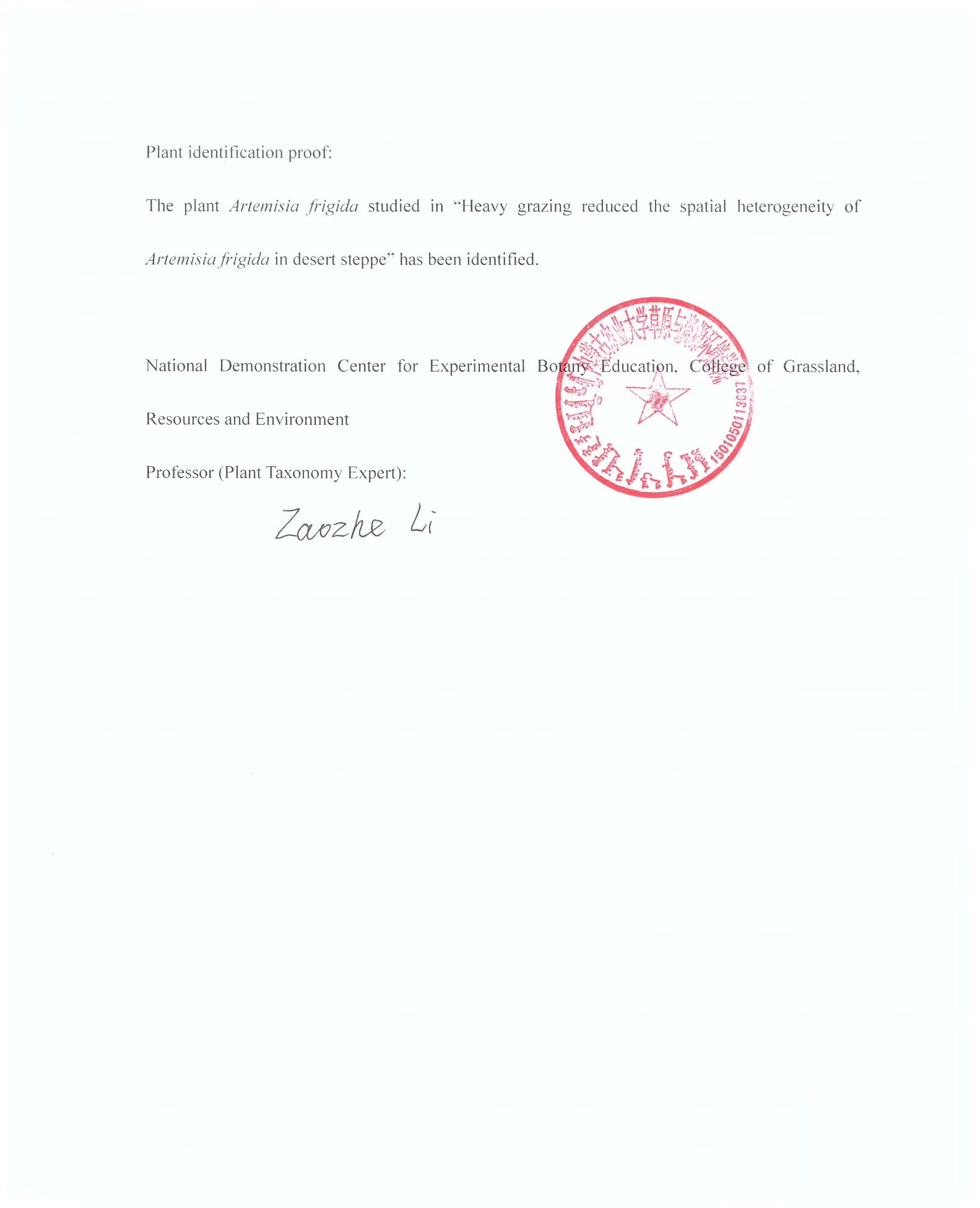

Supplement: Supplementary file 3 — Additional file 3. Plant identification proof. [file 12870_2022_3712_MOESM3_ESM.jpg]

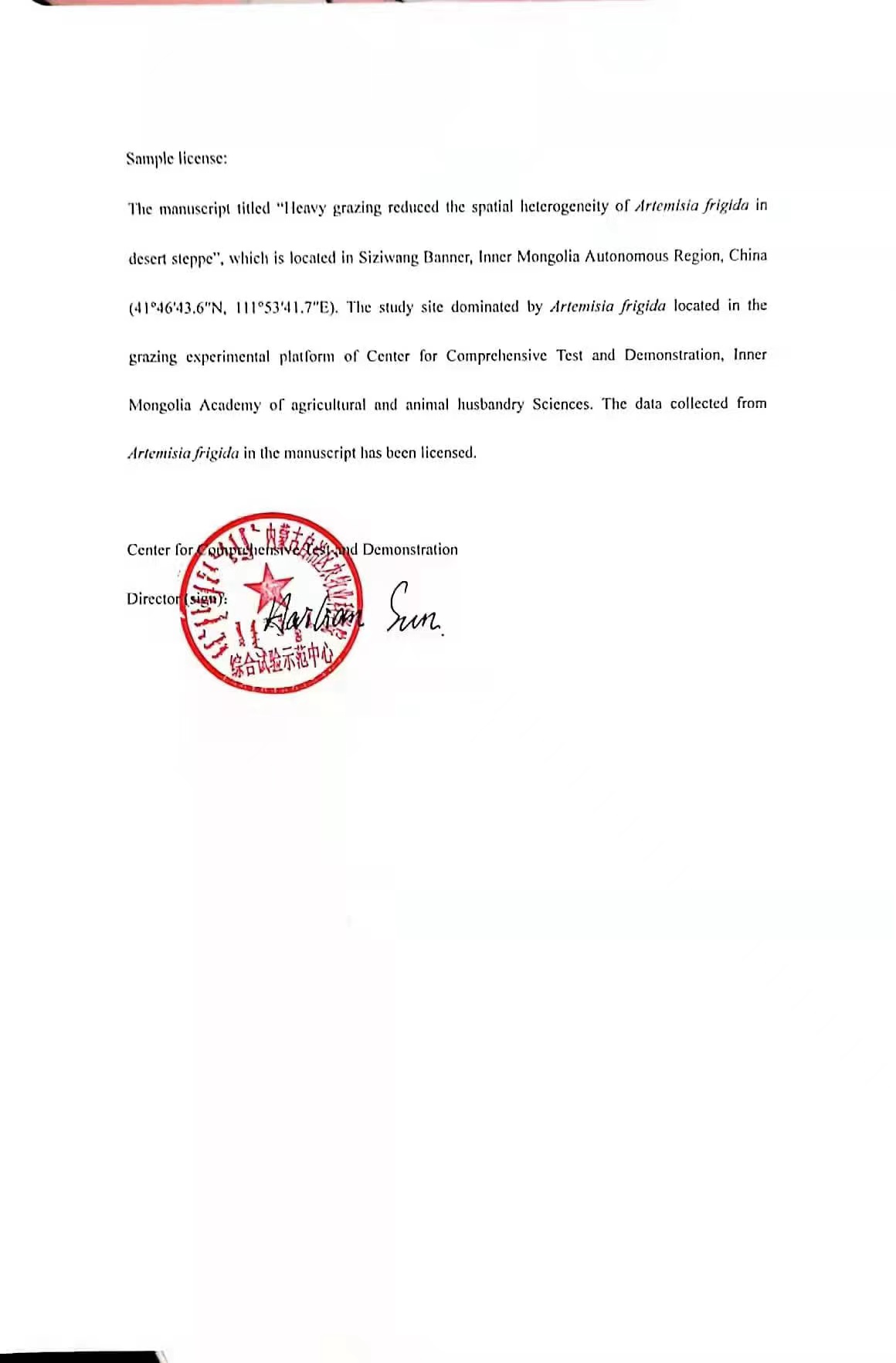

Supplement: Supplementary file 4 — Additional file 4. Sample license. [file 12870_2022_3712_MOESM4_ESM.jpg]
