# Supplementary material for: Heavy grazing reduced the spatial heterogeneity of Artemisia frigida in desert steppe
Source: BMC Plant Biol. 2022 Jul 13;22:337. doi: 10.1186/s12870-022-03712-8 (PMC9281028; doi:10.1186/s12870-022-03712-8)
Supplement: Supplementary file 5 — Additional file 5. Affiliations. [file 12870_2022_3712_MOESM5_ESM.docx]

**Heavy grazing reduced the spatial heterogeneity of *Artemisia frigida* in desert steppe**

Zihan Wang^1^, Shijie Lv^4*^, Guodong Han^1^, Zhongwu Wang^1*^, Zhiguo Li^1^, Haiyan Ren^1^, Jing Wang^1^, Hailian Sun^2^, Guogang Zhang^3^

1 Inner Mongolia Agricultural University, College of Grassland, Resources and Environment, Hohhot 010019, Inner Mongolia, P.R. China.

2 Inner Mongolia Academy of Agricultural and Animal Husbandry Sciences, Hohhot 010010, Inner Mongolia, P. R. China

3 Tianjin Normal University, College of Life Sciences, Tianjin 300387, Tianjin, P.R. China.

4 Inner Mongolia Agricultural University, Science College, Hohhot 010019, Inner Mongolia, P.R. China.

* Corresponding author: Zhongwu Wang, College of Grassland, Resources and Environment, Inner Mongolia Agricultural University, Hohhot, Inner Mongolia, P.R. China. E-mail:wangzhongwu@imau.edu.cn; Shijie Lv, E-mail:lshj123@163.com.
